# Between resistance and adaptation in COVID‐19 times: The outbreak daily prevalence moderates the association between conspiracy thinking and adherence to government protocols

**DOI:** 10.1111/bjhp.70052

**Published:** 2026-01-23

**Authors:** Michele Roccato, Silvia Russo, Moreno Mancosu

**Affiliations:** ^1^ Department of Psychology University of Torino Torino Italy; ^2^ Department of Cultures Politics and Society, University of Torino Torino Italy

**Keywords:** compliance, conspiracy thinking, COVID‐19, multilevel analysis, pandemic prevalence, rolling cross‐section, vaccination

## Abstract

**Objectives:**

Conspiracy thinking has played a significant role in shaping public responses to the COVID‐19 pandemic and has influenced citizens' adherence to government protocols, including reluctance to receive vaccination and adherence to public health measures. However, little attention has been paid to how contextual factors, such as the severity of the pandemic, interact with these beliefs. This study examines how the prevalence of the pandemic, defined as the ratio between the number of cases and swabs each day, moderated the relationship between conspiracy thinking and adherence to public health norms.

**Methods:**

We conducted a secondary analysis of the ResPOnsE COVID‐19 project, a rolling cross‐section two‐wave survey of Italian adults (*N* = 5421) conducted between March and December 2021. Using a multilevel modelling approach, with data nested by day of data collection, we analysed the association between conspiracy beliefs, reluctance to receive a COVID‐19 vaccination and resistance to restrict personal freedoms, interacting conspiracy beliefs with daily variations in pandemic prevalence.

**Results:**

Conspiracy beliefs were positively associated with reluctance to receive the COVID‐19 vaccination and resistance to restrictions. The prevalence of the pandemic moderated these relationships: As the pandemic prevalence increased, conspiracy believers became more reluctant to get vaccinated but more accepting of restrictions on freedom.

**Conclusions:**

Our results emphasize the context‐dependent nature of the consequences of conspiracy beliefs. Rather than uniformly rejecting all public health measures, conspiracist citizens adjust their responses depending on contextual factors. These findings challenge simplistic representations of their attitudes and emphasize the need for dynamic public health communication strategies.

**Statement of Contribution**

**What is already known on this subject?**

- Conspiracy thinking influences compliance with public health measures.
- Previous research has focused on individual psychological predispositions rather than contextual moderators.
- The dynamic interaction between pandemic severity and conspiracy beliefs remains underexplored.

**What does this study add?**

- The effects of conspiracy thinking on compliance are context‐sensitive to epidemiological conditions.
- It highlights the need for adaptive, context‐sensitive public health communication.

## INTRODUCTION

The COVID‐19 pandemic has highlighted the complex relationship between social crises, public health measures and the role of epistemic trust in shaping individual behaviour. A particularly salient factor in this context has been conspiracy thinking, which has been shown to influence compliance with preventive health measures, including vaccine uptake and adherence to restrictions aimed at containing the spread of the virus (Allington et al., 2021; van Prooijen, Etienne, et al., 2023; van Prooijen, Wahring, et al., 2023). While much of the literature on conspiracy beliefs has focused on individual psychological predispositions—such as distrust of institutions, a preference for simplistic causal explanations and cognitive biases—less attention has been paid to how contextual factors can alter the outcomes of these beliefs (for a partial exception, see van Prooijen & Douglas, 2017). Research suggests that conspiracy beliefs do not emerge in a vacuum and that their consequences depend on broader societal conditions, a phenomenon sometimes described as the ‘life cycle of conspiracy theories’ (Mancosu & Vassallo, 2022; see also van Prooijen & Douglas, 2017). Understanding this dynamic is crucial for understanding how conspiracy beliefs translate into behavioural outcomes, especially in times of crisis when public health compliance is essential.

In the context of COVID‐19, conspiracy thinking has appeared in various forms, ranging from the belief that the virus was artificially created to scepticism about the motives behind vaccination campaigns (van Prooijen, Etienne, et al., 2023, van Prooijen, Wahring, et al., 2023). Among other factors, conspiracy thinking is typically associated with a general distrust of institutions and scepticism towards complex or esoteric topics such as science and technology (Swami et al., 2014). This distrust is not merely a form of blind fanaticism, but can be interpreted as a cognitive consequence of individuals' discomfort with scientific uncertainty and complexity (van Prooijen, 2019). The rapid spread of scientific information—and misinformation—during the pandemic has overwhelmed much of the population and led people to rely on the simplistic explanations that conspiracy theories often provide (Freeman et al., 2020). Conspiracy beliefs are also positively related to individuals' need for security and control in times of crisis (van Prooijen & Jostmann, 2013). As a likely result of these factors, some people adopted conspiracy explanations that gave the appearance of understanding and predictability, even when these explanations contradicted scientific evidence (Hornik et al., 2021).

Despite the spread of conspiracy theories, the existence of COVID‐19 has been relatively rarely denied (Roozenbeek et al., 2020), and even those who believe in conspiracies acknowledged the reality of the virus and considered themselves susceptible to infection (Allington et al., 2021). This suggests that conspiracy thinking does not necessarily equate to a complete rejection of all scientific information, but can also reflect scepticism towards certain sub‐narratives or, more generally, towards epistemic authorities. If we conceptualize conspiracy thinking in this way, we can assume that individuals, despite recognizing the threat posed by the virus, may have opted for alternative methods of risk mitigation influenced by their distrust of conventional science and institutions (Earnshaw et al., 2020). For example, some may have viewed official guidelines with suspicion and consequently favoured unproven cures or prioritized personal freedom over collective health measures (Plohl & Musil, 2021), such as reliance on personal protective measures, nutritional supplements or faith‐based interventions (e.g., Arlauskas et al., 2022). While some of these practices may have been beneficial, others provided a false sense of security and directly conflicted with public health recommendations, potentially exacerbating the spread of the virus (World Health Organization, 2020).

The contradiction between recognizing one's own vulnerability to the virus and simultaneously rejecting scientifically endorsed interventions and public health measures (Marinthe et al., 2020) can be explained by considering a basic principle of conspiracy thinking, where distrust of information sources leads to selective acceptance of certain facts while rejecting others (Wood & Douglas, 2015). On one hand, the increasing severity of the crisis may have triggered fear and susceptibility to conspiracy narratives that offer alternative explanations or scapegoats (Van Mulukom et al., 2022). On the other hand, recognition of the seriousness of the situation did not necessarily result in adherence to recommended behaviours, especially when individuals questioned the motives behind these recommendations (Franz & Dhanani, 2021).

## THIS STUDY

### Goal and hypotheses

In this study, we aimed to examine the relationship between conspiracy thinking and health‐related attitudes, such as reluctance to be vaccinated and refusal to accept restrictions on personal freedoms to combat the spread of COVID‐19, with the actual severity of the pandemic as a moderating factor. As the severity of the pandemic increases, individuals experience heightened perceived risk and mortality salience (Pyszczynski et al., 2021). These perceptions can trigger fear‐based and self‐protective motivations that, among conspiracist citizens, do not necessarily foster greater trust in authorities or medical interventions; instead, they may strengthen suspicion towards official accounts (Imhoff & Lamberty, 2020). Nevertheless, such heightened perceptions can prompt pragmatic behavioural adjustments (Marinthe et al., 2020). From this perspective, the daily prevalence of the pandemic may serve as a contextual cue that amplifies existential perceived vulnerability, leading conspiracy‐oriented individuals to prioritize ‘natural’ and controllable measures (e.g., freedom restrictions) while resisting institutionally mandated actions, such as vaccination. In other words, rather than viewing adherents of conspiracy theories as blindly credulous or irrational, we consider the possibility, highlighted in recent psychological literature (e.g., van Prooijen, Etienne, et al., 2023; van Prooijen, Wahring, et al., 2023), that individuals selectively adopt and combine elements of conspiracy theories and scientific information in ways that reflect a relatively nuanced, albeit unconventional, integration of knowledge. This, in turn, has implications for their behavioural responses.

Based on the literature above, we hypothesize that conspiracy thinking is associated with reluctance to receive COVID‐19 vaccination (H1) and rejection of freedom restrictions (H2) (Bruder & Kunert, 2020; Salali & Uysal, 2022). Furthermore, we expect that people who endorse conspiracy theories are even less likely to get vaccinated if the pandemic worsens, as the status of the pandemic might confirm their reluctance, for example, by suggesting that vaccination does not work (H3). However, we expect that, in order to fight and protect themselves from the virus, these individuals might accept milder and more ‘natural’ measures, such as restricting freedom of movement and social interactions (H4).

### Data and methods

We pursued our research objectives through a secondary analysis of Italian data. Italy is particularly interesting regarding public opinion reactions to COVID‐19, as it was the initial epicentre of the outbreak in Europe and paid a dramatically high price for the pandemic (for example, having the most active outbreak in the world in March 2020; see Di Ciaula et al., 2020). Within weeks of the outbreak, the national healthcare system was on the verge of collapse. In the absence of medicines and vaccines, the Italian government had no choice but to lock down citizens for weeks. In the following months, the pandemic followed a fluctuating course, with alternating periods of rising and falling outbreaks (Roccato et al., 2025). For example, after months of relative calm, a new lockdown was imposed in autumn 2020 to counter a strong resurgence of the pandemic.

On 27 December 2020, the first of the new COVID‐19 vaccines was distributed to the population. The Italian public initially reacted enthusiastically to the availability of these vaccines, but in the weeks and months that followed, a negative attitude towards the COVID‐19 vaccines spread and a ‘no‐vax’ movement emerged. In April 2021, 11.7% of Italians were not vaccinated and had no intention of being vaccinated (Roccato & Russo, 2023).

The data we analysed were collected as part of the ResPOnsE COVID‐19 project, a study divided into four waves: (a) 9 April to 2 July 2020 (*n* = 15,757); (b) 21 December 2020 to 2 January 2021 (*n* = 2979); (c) 17 March to 16 June 2021 (*n* = 8210); and (d) 10 November to 22 December 2021 (*n* = 3032). In each wave, a quota sample of the Italian adult population, intended to be representative by gender, age and place of residence, was surveyed online. Further details on the project can be found in Vezzoni et al. (2020, 2022). The data are freely available from the ResPOnsE COVID‐19 website (https://dataverse.unimi.it/dataset.xhtml?persistentId=doi:10.13130/RD_UNIMI/IJDSVS). The ResPOnsE COVID‐19 data are particularly valuable for our research objectives, as they were collected using a rolling cross‐section approach. Each survey day, a subsample of the entire sample was interviewed in the same setting and with the same questionnaire. As the day on which each participant was surveyed was randomly selected, the resulting daily samples were statistically equivalent; therefore, individual responses were nested by the day of the interview (Lutz et al., 2013). We analysed the data using a multilevel approach, with the daily prevalence of the COVID‐19 pandemic as a level‐2 variable (see Cena & Roccato, 2023 and Roccato et al., 2025, for similar approaches). Analyses were conducted using MPLUS 8 (Muthén & Muthén, 1998–2017), estimator MLF. We focused only on the third and fourth waves, as no questions on the intention to be vaccinated against COVID‐19 were available in the first waves. The sample comprised 5421 participants in total. Table 1 shows that the distribution of the sample on the socio‐demographic variables considered substantially overlapped with that of the Italian adult population.

**TABLE 1:** Socio‐demographic comparison between the study sample and the Italian population.

	Sample	Italian population
Women (%)	51.6%	51.2%
Age (mean)	51.5	52.3
Years of education (%)	13.9	12.4
Living in north‐western Italy (%)	28.6%	26.9%
Living in north‐eastern Italy (%)	20.3%	19.6%
Living in central Italy (%)	18.9%	20.0%
Living in southern Italy (%)	21.7%	22.7%
Living in the main Italian islands (%)	10.6%	10.8%

### Measures

We focused our analyses on predicting two dependent variables. First, reluctance to receive the COVID‐19 vaccination, measured by self‐reported vaccine uptake and/or intention to be vaccinated in the future. Participants answered the following four‐category item: ‘Have you been vaccinated against COVID‐19?’ The response options were: 1 = *I have already been vaccinated or, if not yet vaccinated, I will definitely get vaccinated*; 2 = *I have not yet been vaccinated, but will probably get vaccinated*; 3 = *I have not yet been vaccinated and will probably not get vaccinated*; and 4 = *I have not yet been vaccinated and will definitely not get vaccinated*. Second, refusal to restrict one's freedoms to combat the spread of COVID‐19, assessed as the mean of participants’ willingness to restrict their (a) freedom of movement, (b) freedom to meet whomever they want and (c) privacy (i.e., no interference in their private lives). The response options ranged from 0 *Not at all willing* to 10 *Completely willing* and were recoded so that higher scores indicated greater rejection of freedom restrictions. The *α* value for the battery was .859.

The independent variables used were the degree of conspiracy beliefs among participants (level‐1 variable), the actual daily prevalence of the pandemic (level‐2 variable) and their interaction (calculated after standardizing these two variables). Conspiracy beliefs were assessed by averaging the following two Likert items with 11 categories (the extreme categories labelled *Strongly disagree* and *Strongly agree*): ‘Official medicine is too much under the influence of dominant economic interests to develop adequate treatments’ and ‘In Italy, data on the true extent of the pandemic is kept secret’. These items were administered to a randomly selected subsample of the total sample, *n* = 5423. The *α* value for these items was .779. The daily prevalence of the pandemic was assessed as the ratio between the number of cases and the number of swabs each day. The US Center for Disease Control and Prevention considers this indicator one of the key metrics for assessing the impact of COVID‐19 on communities (see CDC, 2025). Moreover, Rahman et al. (2020); see also Millimet and Parmeter (2022) showed that this is the most convincing measure of pandemic severity.

In the analyses, we used participants' gender (0 = *male*, 1 = *female*), age, years of formal education and geopolitical area of residence (*Residence in north‐western Italy*, *Residence in north‐eastern Italy*, *Residence in central Italy* and *Residence in southern Italy*; *Residence in the main Italian islands* was used as the reference category) as level‐1 control variables. To rule out the alternative interpretation that our results depend on the mere passage of time rather than a true association between the severity of the pandemic and our dependent variables, we controlled for the effect of the wave of data collection by including it as a level‐2 control variable. Table 2 presents the descriptive statistics for the variables used and their bivariate correlations.

**TABLE 2:** Descriptive statistics for the study variables and bivariate correlations between them.

	Mean	*SD*	2.	3.	4.	5.	6.	7.	8.	9.	10.	11.	12.	13.
*Level 1*														
1. Woman	48.44	.50	−.06***	−.04**	−.03**	−.03**	.03**	−.02	.07***	.09***	.06***	.04**	.04**	−.00
2. Age	51.51	15.87		.16***	.06***	−.03*	.01	−.05***	.05***	−.03	−.11***	.13***	.12***	.17***
3. Years of education	13.91	3.02			.01	−.04**	.03*	.02	−.18***	−.17***	−.08***	.01	.01	−.02
4. Living in north‐western Italy	.29	.45				−.90***	−.89***	−.91***	−.05***	−.05***	−.01	.01	.01	.03
5. Living in north‐eastern Italy	.21	.40					−.85***	−.87***	−.02	.00	.06***	−.05***	−.04**	−.03*
6. Living in central Italy	.19	.39						−.86***	−.02	−.05***	−.04**	−.01	−.01	−.03
7. Living in southern Italy	.22	.41							−.08***	.07***	−.03	.04**	.04*	.02
8. Conspiracy 1	5.48	3.14								.64***	.28***	−.01***	−.10***	−.09***
9. Conspiracy 2	4.28	3.45									.34***	−.12***	−.12***	−.12***
10. Reluctance to receive vaccination	1.37	.83										−.23***	−.21***	−.22***
11. Reluctance of restrictions 1	5.93	3.08											.81***	.63***
12. Reluctance of restrictions 2	6.03	3.09												.58***
13. Reluctance of restrictions 3	5.50	3.3												
*Level 2*														
1. Wave of data collection (1 = fourth wave)	.53	.50	−.79***											
2. Pandemic prevalence	1.04	.87												

*Note*: Conspiracy 1: ‘Official medicine is too much under the influence of dominant economic interests to develop adequate treatments’. Conspiracy 2: ‘In Italy, data on the true extent of the pandemic is kept secret’. Reluctance to restrictions 1: ‘To stop the spread of COVID‐19, how willing are you to limit the following personal freedoms for yourself? Freedom of movement’. Reluctance to restrictions 2: ‘To stop the spread of COVID‐19, how willing are you to limit the following personal freedoms for yourself? Freedom to meet whoever you want’. Reluctance to restrictions 3: ‘To stop the spread of COVID‐19, how willing are you to limit the following personal freedoms for yourself? Privacy (i.e., the freedom not to have interference in your private life)’. For dummy variables, the ‘mean’ is the proportion, on a 0–1 scale, of the 1 category. When a dummy and a cardinal variable are involved, we report their point‐biserial correlation. When two dummy variables are involved, we report their polychoric correlation.

***p < .001, **p < .01, *p < .05.

### Results

Two preliminary unconditional models showed that the two dependent variables varied significantly across days of data collection. For vaccination hesitancy, the between‐level variance was .013, *p* < .002, ICC = .019; for unwillingness to restrict one's freedoms to combat the spread of COVID‐19, it was .108, *p* < .024, ICC = .014. These significant variations justified the use of multilevel modelling.

**FIGURE 1:**
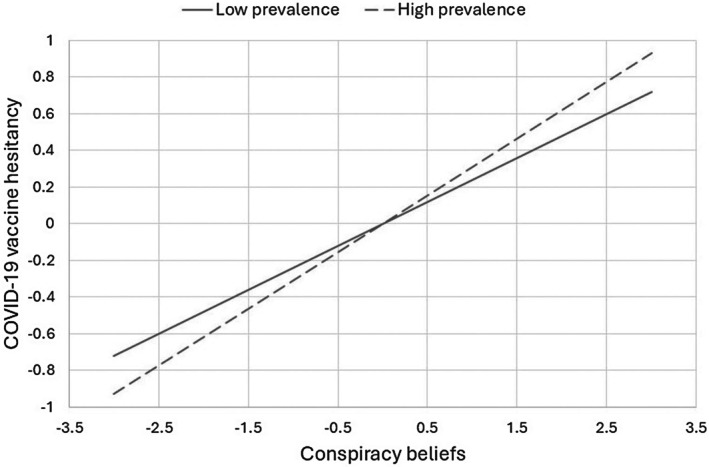
The daily prevalence of the pandemic moderates the association between conspiracy beliefs and COVID‐19 vaccine hesitancy.

Table 3 (first three columns) presents the results of the model predicting vaccination aversion. Among the control variables, being a woman was positively associated with reluctance to receive COVID‐19 vaccination, while age, residence in southern Italy and being interviewed in the fourth wave of data collection were negatively associated with it. More relevant to our objectives, and consistent with H1, conspiracy beliefs were positively associated with reluctance to receive COVID‐19 vaccination. Daily prevalence of the pandemic was not directly associated with reluctance, but it moderated the association between conspiracy beliefs and reluctance to receive COVID‐19 vaccination. Consistent with H3, a simple slope analysis showed that the association between conspiracy beliefs and reluctance to receive COVID‐19 vaccination was weaker when the daily prevalence of the pandemic was low (−1 *SD*), *b* = .240, *SE* = .026, *p* < .001, and stronger when the daily prevalence was high (+1 *SD*), *b* = .310, *SE* = .03, *p* < .001. In other words, when the pandemic situation was severe, individuals who believed in conspiracies were more reluctant to get vaccinated. Figure 1 graphically illustrates this moderated association.

**TABLE 3:** Prediction of vaccine hesitancy and of willingness to limit one's own personal liberties (unstandardized parameters are displayed).

	COVID‐19 vaccine hesitancy			Unwillingness to limit one's own freedom		
	*b*	*SE*	*p*	*b*	*SE*	*p*
Gender (1 = woman)	.050	.025	.042	−.331	.092	<.001
Age	−.006	.001	<.001	−.032	.003	<.001
Education	−.006	.004	.139	.008	.014	.717
Living in north‐western Italy	.008	.041	.853	.221	.191	.248
Living in north‐eastern Italy	.076	.042	.070	.431	.167	.010
Living in central Italy	−.047	.047	.316	.330	.201	.100
Living in southern Italy	−.108	.042	.010	−.109	.190	.564
Wave of data collection	−.158	.050	.001	.580	.184	.002
Conspiracy beliefs	.275	.018	<.001	.460	.047	<.001
Prevalence of the pandemic	.028	.037	.445	−.154	.102	.131
Conspiracy beliefs*Prevalence of the pandemic	.035	.017	.043	−.088	.039	.026

**FIGURE 2:**
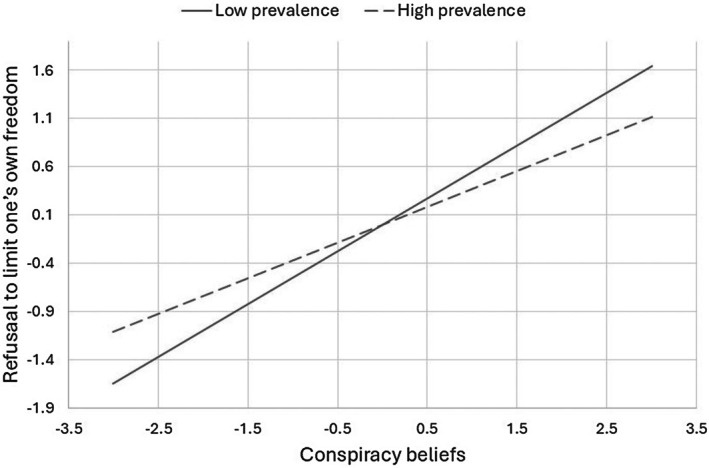
The daily prevalence of the pandemic moderates the association between conspiracy beliefs and willingness to limit one's own liberties.

The second three columns of Table 3 present the results of the model predicting refusal to restrict one's freedoms. Being a woman and age were negatively associated with this variable, while living in north‐western Italy and the wave of data collection were positively associated with it. More relevant to our objectives, and consistent with H2, conspiracy beliefs were positively associated with aversion to restricting one's freedoms, while daily prevalence of the pandemic was not associated with it but moderated the former association. A simple slope analysis showed that, consistent with H4, the association between conspiracy beliefs and reluctance to restrict one's freedoms was stronger when the daily prevalence of the pandemic was low (−1 *SD*), *b* = .548, *SE* = .064, *p* < .001, than when it was high (+1 *SD*), *b* = .372, *SE* = .059, *p* < .001. In other words, when the pandemic was severe, individuals who believed in conspiracies were less likely to reject restrictions on freedom. Figure 2 graphically depicts this moderated association. Overall, the results suggest that people who endorsed conspiracy theories were more reluctant to get vaccinated when the pandemic was severe but were more willing to restrict their freedom to contain the spread of the virus.

## DISCUSSION AND CONCLUSION

Vaccination is the most cost‐effective tool available to prevent viral and bacterial outbreaks (Heinrich et al., 2024). However, during pandemics, access to all necessary medical countermeasures and vaccines is not sufficient: public participation is essential and individuals must actively contribute to crisis management (Schaffer et al., 2020). In this context, the response to a pandemic is based on a social contract (Kreps & Kriner, 2023). This has been the case in the past, for example, in Venice during the leprosy outbreak and in England during the Black Death (Newman, 2012) and remains relevant today, even with advances in science and medicine. Therefore, it is important to understand which factors encourage or discourage people from adhering to necessary, albeit challenging and costly, measures.

In this study, we investigated how conspiracy beliefs about COVID‐19 relate to adherence to measures such as vaccination and restrictions on freedom to contain the spread of the virus. Consistent with previous literature (Bruder & Kunert, 2020; Salali & Uysal, 2022), our results confirmed that people who believe in conspiracies are more reluctant to be vaccinated and to accept restrictions on their freedom for the common good. Interestingly, we also found that these associations change with fluctuations in the severity of the pandemic. When the pandemic is more severe, that is, on days of higher prevalence, conspiracy theorists are more likely to oppose vaccination, a drug‐based solution that has also been heavily criticized in certain sections of public opinion. At the same time, they are more willing to accept restrictions on their freedom, a milder and more ‘natural’ solution. These patterns of reaction to the severity of the pandemic suggest that conspiracy theorists are sensitive to current events and counter this vulnerability by favouring more natural containment strategies.

Obviously, the prevalence of COVID‐19 at the population level inevitably overlaps with other variables. For example, the literature shows that the more severe the pandemic, the more extensive the searches for terms such as ‘coronavirus’, ‘COVID‐19’ and ‘COVID’ on Google (Saegner & Austys, 2022), as well as the volume of media coverage and government declarations on the outbreak (Satpathy et al., 2021). Although our data did not allow us to disentangle the specific role of these variables, we presume that all of them may contribute to a heightened perception of threat and, consequently, to fear‐ and control‐based mechanisms that prompt conspiracy believers to embrace natural solutions rather than accept medical ones.

This work has led to at least two important contributions. First, our findings reinforce the idea that the COVID‐19 pandemic, even after it has fully unfolded, remains a uniquely valuable period for studying the mechanisms underlying conspiracy beliefs. The crisis created an environment of extreme uncertainty, high information volatility and intense public debate, providing an unprecedented opportunity to observe how individuals construct and adapt their belief systems in real time. Second, we have demonstrated the importance of integrating contextual factors and individual conspiracy beliefs—an approach that remains relatively rare in the literature. The notion that conspiracy theories and their associated ideas do not emerge or persist in a vacuum, but rather fluctuate as a function of broader social and informational dynamics, is occasionally present in the literature (see Mancosu & Vassallo, 2022; van Prooijen & Douglas, 2017). According to this perspective, conspiracy beliefs and their correlated constructs are in flux over time, influenced by changes in the climate of opinion, political events and perceived crises. Notably, we have shown that the relationship between conspiracy beliefs and public health behaviours depends on the severity of the pandemic, highlighting how external conditions can influence the behavioural consequences of conspiracy thinking. More specifically, our findings contribute to contemporary debates in conspiracy research by showing that conspiracy thinking does not lead to uniform or purely oppositional responses to public health measures. Rather, it can shape behaviour in counterintuitive ways, steering individuals towards what they perceive as consistent with their ideological viewpoint. This finding aligns with recent literature arguing that conspiracy thinking is not a monolithic or entirely irrational way of thinking, but rather the outcome of a selective process in which individuals choose elements of different narratives that make sense within their broader worldview (see van Prooijen, Etienne, et al., 2023; van Prooijen, Wahring, et al., 2023).

The secondary analysis approach we used resulted in some limitations in this study, mainly related to the sample, the available measures and the study design. Regarding the sample, the study was based on a quota sample of the adult population surveyed online from a single country. Therefore, it is likely that individuals with limited internet access and those uncomfortable with online surveys were under‐represented in our study (MacInnis et al., 2018). Although Italy was an ideal context for our research purposes, the single‐country approach precludes straightforward generalization of our findings to other countries. Overall, however, the quality of our sample was much higher than that of the convenience samples typically used in psychological research. According to some authors (e.g., Wang et al., 2015), the quality of data collected from probability‐based samples can no longer be distinguished from that of data obtained from non‐probability‐based online samples, due to the ever‐decreasing response rates to traditional surveys. Nevertheless, replicating this study using stratified samples from diverse countries could be of interest to determine whether these patterns of results also hold in contexts that were less affected by the pandemic or where the vaccination campaign was conducted differently. For example, previous studies have shown that conspiratorial ideas, especially the most extreme ones, are slightly more prevalent in countries where government COVID‐19 risk communication started later than at the time of discovery of the first COVID‐19 case (Chan et al., 2021). This suggests that differences in how governments have handled communication about the COVID‐19 pandemic and the administration of the vaccine should be given special consideration in future research.

Second, we had to rely on self‐reported short measures. Surveys on socially relevant topics may prompt participants to provide socially acceptable responses. In our case, this could have led them to underreport their actual level of conspiracy beliefs and overreport their adherence to health measures. However, online surveys typically result in significantly less social desirability bias than traditional survey methods (e.g., Triga & Manavopoulos, 2019). Moreover, the measure of conspiracy beliefs included only two items relating to belief in economic interests behind official medicine and the secrecy of true pandemic data. Substantively, pandemic‐related conspiracy beliefs are much broader in content and different beliefs have different effects on behaviour. For example, Imhoff and Lamberty (2020) showed that conspiracy beliefs portraying the pandemic as a hoax were associated with lower adherence to containment measures, while theories assuming sinister forces intentionally created the virus were associated with increased precautionary behaviours, such as using alternative remedies or hoarding. Methodologically, it is likely that our two‐item measure was less reliable than a multi‐item measure. Against this background, our findings may have underestimated the actual strength of the relationships we focused on in this study. Future studies examining a broader range of conspiracy beliefs and including a social desirability scale, such as Paulhus' (1984) Balanced Inventory of Socially Desirable Responding, could provide conceptually important and statistically stronger insights into how certain beliefs relate to adherence to restrictions and willingness to vaccinate, depending on the severity of the pandemic.

Finally, due to the rolling cross‐sectional approach of the dataset, different people were surveyed each day. Although, strictly speaking, we could not determine whether individual beliefs and behaviours changed in response to the daily prevalence data, rolling cross‐sectional studies move survey research closer to true causal inference (Johnson & Brady, 2002). However, a true longitudinal replication of this study, tracking the same individuals over time, would be needed to make even stronger claims about causality.

### These limitations should not overshadow the strengths of the paper

First, our sample allowed for a cautious generalization of our findings to the entire Italian population; thus, our study was characterized by strong external validity. Second, we demonstrated the importance of adopting a social‐psychological perspective that considers both individual (conspiracy beliefs) and contextual (severity of the pandemic) factors when examining compliance. Researchers have widely emphasized that psychological factors are the primary determinants of preventive behaviours (e.g., Giancola, 2022; Hornsey et al., 2018; Scrima et al., 2022). Although these factors are important, they need to be considered in context: In this study, we showed how the fluctuations of the pandemic can modulate the relationship between psychological predispositions and behavioural intentions.

### We believe that our findings may have concrete public health implications

Previous literature shows that during periods of lower severity, prebunking—brief inoculation‐style interventions—should be used to build resistance to misinformation before anxiety and rumour volumes increase (Roozenbeek et al., 2022). As severity rises, messaging should remain autonomy‐supportive by acknowledging concerns, providing a clear rationale and offering concrete, feasible options, rather than relying on controlling or moralizing frames (see Legate et al., 2022). It is therefore unsurprising that these approaches are effective: They treat conspiracist citizens not as naïve or intransigent, but as actors whose risk management responds to contextual cues—precisely as our results suggest.

Overall, our findings contribute to models of infection prevention and vaccination behaviour by showing that the effects of conspiracy thinking on compliance are not static but are context‐sensitive reactions shaped by epidemiological conditions. This evidence supports a dynamic perspective on health behaviour, in which both adherence to and resistance against preventive measures depend on situational cues, perceived risk and information environments. For policy and practice, these results suggest that public health communication should adapt to changing levels of outbreak severity. From this perspective, citizens who endorse conspiracy beliefs should not be seen as unresponsive or irrational, but as contextually responsive actors whose health behaviours can be influenced through informed communication strategies.

## AUTHOR CONTRIBUTIONS

**Michele Roccato:** Conceptualization; methodology; formal analysis; writing – original draft; writing – review and editing. **Silvia Russo:** Conceptualization; formal analysis; writing – original draft; writing – review and editing. **Moreno Mancosu:** Conceptualization; writing – original draft; writing – review and editing.

## FUNDING INFORMATION

This research received no specific grant from any funding agency, commercial or not‐for‐profit sectors.

## CONFLICT OF INTEREST STATEMENT

The authors declare none.

## ETHICS STATEMENT

The authors assert that all procedures contributing to this work comply with the ethical standards of the relevant national and institutional committees on human experimentation and with the Helsinki Declaration of 1975, as revised in 2008.

## Data Availability

The data that support the findings of this study are available in UNIMI Dataverse at https://dataverse.unimi.it/. These data were derived from the following resources available in the public domain: ‐ ResPOnsE COVID‐19. Cumulative file: Wave 1 to Wave 4, https://doi.org/10.13130/RD_UNIMI/FF0ABQ.
